# Down-Regulation of *ECRG4,* a Candidate Tumor Suppressor Gene, in Human Breast Cancer

**DOI:** 10.1371/journal.pone.0027656

**Published:** 2011-11-16

**Authors:** Renaud Sabatier, Pascal Finetti, José Adelaide, Arnaud Guille, Jean-Paul Borg, Max Chaffanet, Lydie Lane, Daniel Birnbaum, François Bertucci

**Affiliations:** 1 Département d'Oncologie Moléculaire, Centre de Recherche en Cancérologie de Marseille, UMR891 INSERM and Institut Paoli-Calmettes Marseille, Marseille, France; 2 Département d'Oncologie Médicale, Centre de Recherche en Cancérologie de Marseille, Institut Paoli-Calmettes Marseille, Marseille, France; 3 Université de la Méditerranée, Marseille, France; 4 Département de Polarité cellulaire, signalisation et cancer, Centre de Recherche en Cancérologie de Marseille, U891 INSERM and Institut Paoli-Calmettes Marseille, Marseille, France; 5 SIB-Swiss Institute of Bioinformatics, Geneva, Switzerland; 6 Department of Human Protein Science, University of Geneva, Geneva, Switzerland; Institut de Génomique Fonctionnelle de Lyon, France

## Abstract

**Introduction:**

*ECRG4/C2ORF40* is a potential tumor suppressor gene (TSG) recently identified in esophageal carcinoma. Its expression, gene copy number and prognostic value have never been explored in breast cancer.

**Methods:**

Using DNA microarray and array-based comparative genomic hybridization (aCGH), we examined *ECRG4* mRNA expression and copy number alterations in 353 invasive breast cancer samples and normal breast (NB) samples. A meta-analysis was done on a large public retrospective gene expression dataset (n = 1,387) in search of correlations between *ECRG4* expression and histo-clinical features including survival.

**Results:**

*ECRG4* was underexpressed in 94.3% of cancers when compared to NB. aCGH data revealed *ECRG4* loss in 18% of tumors, suggesting that DNA loss is not the main mechanism of underexpression. Meta-analysis showed that *ECRG4* expression was significantly higher in tumors displaying earlier stage, smaller size, negative axillary lymph node status, lower grade, and normal-like subtype. Higher expression was also associated with disease-free survival (DFS; HR = 0.84 [0.76–0.92], *p* = 0.0002) and overall survival (OS; HR = 0.72 [0.63–0.83], *p* = 5.0E-06). In multivariate analysis including the other histo-clinical prognostic features, *ECRG4* expression remained the only prognostic factor for DFS and OS.

**Conclusions:**

Our data suggest that *ECRG4* is a candidate TSG in breast cancer, the expression of which may help improve the prognostication. If functional analyses confirm this TSG role, restoring *ECRG4* expression in the tumor may represent a promising therapeutic approach.

## Introduction

Breast cancer is the most frequent and deadly cancer in women in Western countries. Despite the mass screening and multidisciplinary therapeutic progresses, a substantial number of patients (∼25%) die from metastatic disease. Breast cancer is a complex disease characterized by the accumulation of multiple molecular alterations, genetic and epigenetic, which disturb the expression of genes controlling critical regulatory processes. Efforts have been directed at the identification of genes that play important roles in mammary oncogenesis and metastatic processes and that could represent new therapeutic and/or prognostic targets. Key genes have been identified, including oncogenes encoding hormone receptors (ER and PR) and tyrosine kinase receptors (ERBB2, EGFR), and tumor suppressor genes (TSG) such as *TP53*, *BRCA1*, and *BRCA2.* However, our molecular understandings of breast cancer, together with clinical benefits for patients, remain limited.

Esophageal cancer-related gene 4 (*ECRG4*), officially called *C2ORF40*, was cloned and identified from normal esophageal epithelium [1]. It is localized in 2q12.2. The encoded protein (augurin) is a secretory molecule produced in endocrine tissues such as pituitary gland, adrenal gland and choroid plexus [2]. Its actions consist in cerebrospinal fluid homeostasis, stimulation of neuroprogenitor cells after brain injury [3], and induction of cell senescence in central nervous system [4]. Even if its impact on oncogenesis is not clear, it has been described as a putative TSG in several cancers including esophageal squamous cell carcinoma [5]-[9], prostate cancer [10], colo-rectal cancer and glioma [8], [11]. *ECRG4* expression was associated with better survival in esophageal [6] and prostate [10] carcinomas, and with inhibition of cell proliferation and migration in esophageal cancer [7]-[9], colorectal cancer and glioma [9], [11]. Surprisingly, no data are available regarding *ECRG4* expression in breast cancer.

Here, we have analyzed the expression of *ECRG4* in a large series of breast cancers profiled using DNA microarrays and its correlation with histo-clinical features and survival.

## Materials and Methods

### Ethics statement

The study was approved by our institutional review board: the Institut Paoli Calmettes (IPC) “Comité d'Orientation Stratégique”. Each patient gave a written informed consent for research use.

### Gene expression data

To determine *ECRG4* mRNA expression in breast cancer and normal breast, we first analyzed gene expression data generated by our laboratory (IPC, Marseille, France) from cancer and normal mammary samples. Tumor tissues were from 353 patients with invasive adenocarcinoma who underwent initial surgery at IPC between 1987 and 2007. Samples were macrodissected and frozen in liquid nitrogen within 30 min of surgical removal. All profiled specimens contained more than 60% of cancer cells (as assessed before RNA extraction using frozen sections adjacent to the profiled samples). After surgery, patients received an adjuvant multimodal treatment according to standard guidelines. Extraction of nucleic acids from frozen samples was done by using guanidium isothiocynanate and cesium chloride gradient as described previously [12]. RNA integrity was controled on Agilent Bioanalyzer (Agilent Technologies®, La Jolla, CA, USA). We had also profiled 4 normal breast (NB) tissue samples, which represented 1 pool of 4 samples from 4 healthy women (reduction mammoplasty), and 3 commercial pools of respectively 1, 2 and 4 normal breast RNA (Clontech, Palo Alto, CA). Expression profiles had been established for these 353 cancers and 4 NB pools with Affymetrix U133 Plus 2.0 human microarrays (Affymetrix®, Santa Clara, CA, USA) as previously described [13]. All data are MIAME compliant and the raw data have been deposited in the MIAME-compliant GEO database (GSE23720, GSE21653, GSE17987 and GSE31448).Data were analyzed by the Robust Multichip Average method [14] in R using Bioconductor and associated packages. *ERCG4* expression was measured by analyzing the sole Affymetrix probe set present, ID 223623_at, the specificity of which was verified using the NCBI program BLASTN 2.2.25+ (**Table S1**). Before analysis, expression level for each tumor was centered by the average expression levels of the four NB samples. Data were then log2-transformed for analysis and display.

To examine the correlation between *ECRG4* mRNA expression and histo-clinical features of tumors in a large series, we pooled our data set with 5 publicly available data sets comprising at least one probe set representing *ECRG4*. These sets were collected from the National Center for Biotechnology Information (NCBI)/Genbank GEO database (series entry GSE1456 [15], GSE3494 [16], GSE4922 [17], GSE6861/GSE4779 [18]) or at the following web address https://genome.unc.edu/pubsup/breastGEO/(Table **S1**). This resulted in a total of 1,387 invasive breast cancers with *ECRG4* mRNA expression and histo-clinical data available for meta-analysis (**Table 1**). To be comparable across data sets and to exclude bias from population heterogeneity, *ECRG4* expression levels were standardized within each data set using the luminal A population as reference. The intrinsic molecular subtypes of tumors were defined as previously described [19] using the Single Sample Predictor (SSP) classifier based on a list of 306 intrinsic genes [20].

**Table 1:** Histo-clinical characteristics of the 1,387 breast cancer patients.

Characteristics	N (%)
Sex	
Female	1387 (100%)
Age (years)	
≤50	380 (37%)
>50	637 (63%)
Histological type	
DUC	509 (82%)
LOB	32 (5%)
MIX	28 (4%)
MED	24 (4%)
Other	31 (5%)
Clinical stage	
I	86 (29%)
II	138 (46%)
III	55 (19%)
IV	18 (6%)
pN	
Negative	460 (43%)
Positive	619 (57%)
pT	
pT1	320 (31%)
pT2	517 (50%)
pT3	169 (16%)
pT4	37 (3%)
SBR Grade	
1	172 (13%)
2	475 (37%)
3	631 (49%)
ER (IHC)	
Negative	499 (44%)
Positive	624 (56%)
PR (IHC)	
Negative	364 (50%)
Positive	363 (50%)
ERBB2 (IHC)	
Negative	261 (72%)
Positive	100 (28%)
Relapse*	
No	755 (67%)
Yes	365 (33%)
5-year DFS*	68%
Death*	
No	544 (73%)
Yes	199 (27%)
5-year OS*	80%
pCR	
No	96 (58%)
Yes	70 (42%)

N, number of cases available; DUC, ductal carcinoma, LOB, lobular carcinoma, MIX, mixed; MED, medullary carcinoma; pN, pathological lymph node involvement; pT, pathological tumor size; IHC, immunohistochemistry; ER, estrogen receptor; PR, progesterone receptor; DFS, disease-free survival; OS, overall survival;

*, non-stage IV patients; pCR, pathological complete response to primary chemotherapy defined as disappearance of the invasive component of the primary tumor after treatment.

To attempt exploring the biological pathways linked to *ECRG4* expression, we identified genes correlated with *ECRG4* mRNA levels using Significance Analysis of Microarrays (SAM) [21] in our own data set. We compared the 50 tumors with the highest expression level to the 50 tumors with the lowest one. We applied a Δ-value of 2.4 and a false discovery rate of 0.1%. Ontology analysis of the resulting gene list was performed using Ingenuity Pathway Analysis (IPA) software (Redwood City, CA, USA) [22]. We only studied pathways with at least 10 genes represented, and with a *p*-value lower than 0.01.

### Array-comparative genomic hybridization data

We analyzed data on genomic imbalances for 247 out of the 353 breast tumors, generated by array-comparative genomic hybridization (aCGH) using 244K CGH Microarrays (Hu-244A, Agilent Technologies) as previously described [12]. A pool of 13 normal male DNA had been used as reference. Extraction of data (log_2_ ratio) was done from CGH Analytics, whereas normalized and filtered log_2_ ratio was obtained from “Feature Extraction” software (Agilent Technologies). The *ECRG4* locus at 2q12.2 was analyzed and copy number changes were characterized as reported previously [12]. Three probes (A_16_P15770886, A_14_P201475, A_14_P138926) matched the *ECRG4* gene on our Agilent chips.

### Statistical analyses

Comparison of mean *ECRG4* mRNA expression level according to classical histo-clinical factors was done using Student t-test (2 variables) or one-way analysis of variance (ANOVA; more than 2 variables). Disease-free survival (DFS) was calculated from the date of diagnosis until date of relapse or death when date of relapse was not available. Overall survival (OS) was calculated from the date of diagnosis to the date of death from breast cancer. Follow-up was measured from the date of diagnosis to the date of last news for patients without event. Survivals were calculated using the Kaplan-Meier method and curves were compared with the log-rank test. Univariate and multivariate survival analyses were done using Cox regression analysis (Wald test). Variables tested in univariate analyses included patients' age at time of diagnosis (≤50 years *vs* >50), pathological tumor size (pT: pT1 *vs* pT2-4), pathological axillary lymph node status (pN: negative *vs* positive), pathological grade (I *vs* 2-3), immunohistochemical (IHC) estrogen receptor (ER), progesterone receptor (PR), and ERBB2 status (negative *vs* positive), histological type, and *ECRG4* expression (continuous value). Variables with a p-value <0.01 in univariate analysis were tested in multivariate analysis. All statistical tests were two-sided at the 5% level of significance. Statistical analysis was done using the survival package (version 2.30) in the R software (version 2.9.1; http://www.cran.r-project.org/). We followed the reporting REcommendations for tumor MARKer prognostic studies (REMARK criteria) [23].

## Results

### *ECRG4* mRNA expression in breast cancer

**Figure 1:**
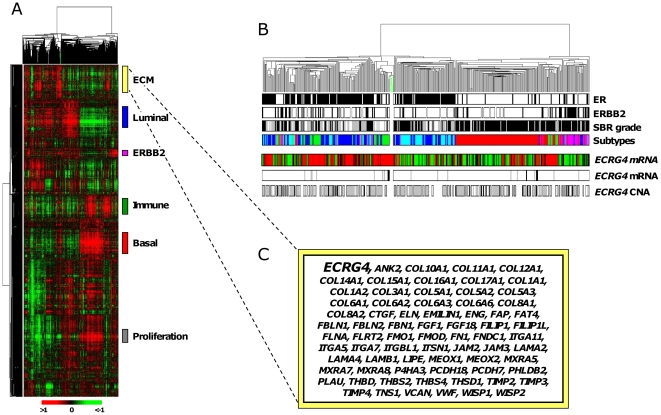
mRNA expression of *ECRG4* in breast cancer. (A) Thumbnail of the hierarchical clustering of the 353 breast cancers and 4 NB samples (columns) and the 12,304 most variable genes (rows). According to a log_2_ pseudocolor scale (bottom), red indicates a high level of mRNA expression compared to the median value across all samples, whereas green indicates a low level of expression. The magnitude of deviation from the median is represented by the colour saturation. The dendrogram of samples (above matrixes) represents overall similarities in gene expression profiles and is zoomed in B. Green branches indicate the 4 NB samples. To the right of the color matrix, are represented some biologically relevant gene clusters. The extra-cellular matrix (ECM)-related cluster, which includes *ECRG4*, is detailed in C. (B) Samples dendogram. Green branches indicate the 4 NB samples. Under the dendogram are reported some histo-clinical tumor features colored as below: ER IHC status (white, negative, and black, positive); ERBB2 IHC status (white, negative, and black, positive); SBR Grade (white, 1, grey, 2; and black, 3molecular subtypes (dark blue, luminal A, light blue, Luminal B, pink, ERBB2, red, basal-like, and green, normal-like). Some molecular features regarding *ECRG4* are represented below: mRNA expression level (median-centered and color-coded as in A), expression status as compared to NB (overexpression, black, neutral, grey, and underexpression, white), and aCGH-based copy number alteration (CNA: gain, black, neutral, grey, and loss, white). (C) Details of the genes belonging to the ECM gene cluster.

We first analyzed expression data generated in our laboratory by using Affymetrix microarrays from 357 mammary samples including 353 pre-treatment primary cancers and 4 NB samples. Compared to NB, 333 tumors (94.3%) showed underexpression (defined by ratio T/NB ≤0.66), whereas only 5 tumors (1.4%) showed overexpression (ratio T/NB >1.5), and 15 (4.2%) showed similar expression (0.66< ratio T/NB ≤1.5). Whole-genome hierarchical clustering showed that *ECRG4* was located within an archetypal extracellular matrix-related gene cluster, including for example several collagen, integrin and metalloproteinase genes (**Figure 1**).

Data from aCGH were available for 247 of the 353 tumor samples from our institution, allowing us to analyze the *ECRG4* locus at 2q12.2. Loss/deletion of this region has not been reported as recurrent in breast cancer. In our series, a DNA copy number alteration (1.5 fold change as compared to normal DNA) was present in 10 tumors (10%) for the gains, and 44 (18%) for the losses, and absent in 179 tumors (72%). There was no significant difference in the frequency of *ECRG4* copy number alteration between the molecular subtypes (p = 0.08, Fisher's exact test).

Regarding the DNA/RNA correlations, 44 out of the 44 (100%) tumors with DNA loss showed mRNA underexpression; however, 23 out of the 24 (96%) tumors with DNA gain and 172 out of the 179 (96%) tumors with “normal” DNA copy number also showed underexpression, suggesting that *ECRG4* loss is not the main mechanism of underexpression in breast cancer.

### *ECRG4* expression and histo-clinical correlations

**Figure 2:**
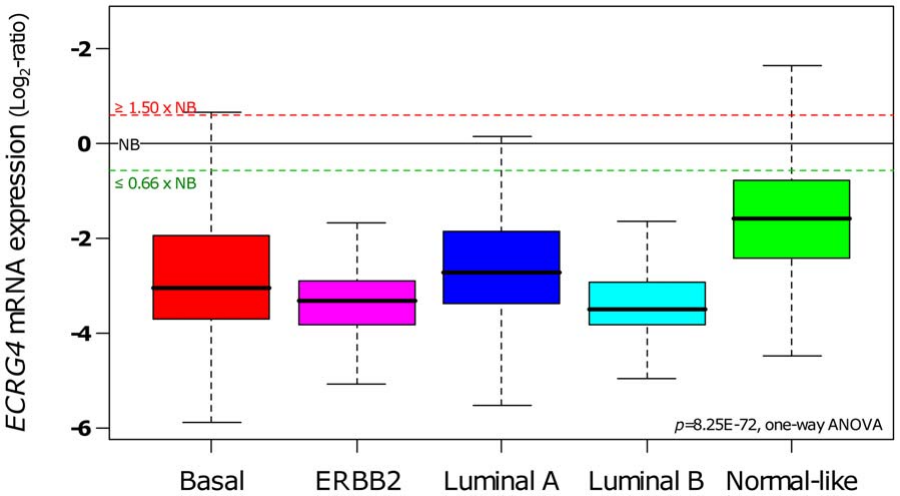
mRNA expression of *ECRG4* according to breast cancer molecular subtypes. *ECRG4* expression across 1,387 breast cancer samples was examined according to molecular subtypes. Box plots of *ECRG4* expression are shown according to basal, ERBB2, luminal A, luminal B, and normal-like subtypes. Expression values are NB-centered. The horizontal black line represents the level of expression of *ECRG4* in normal breast (NB) tissue. Differences in *ECRG4* expression levels between the subtypes were tested for significance using one-way ANOVA. For each box plot, median and ranges are indicated.

We searched for correlations between *ECRG4* mRNA expression and histo-clinical features of tumors in a large data set of 1,387 invasive breast cancers, including our series and 5 public microarray data sets. Of note, the pattern of expression was observed homogeneously through all the data sets (**Figure S1**), and more than 90% of tumor samples showed *ECRG4* underexpression as compared to NB in each data set and in the pooled data set. As shown in **Table 2**, *ECRG4* expression was significantly (t-test) associated with age inferior to 50 years, early clinical stage, small pathological tumor size, absence of axillary lymph node involvement, low tumor grade, and histological type (being the highest in lobular type and the lowest in medullary type). No significant association was found with IHC expression of ER, PR and ERBB2. Regarding the molecular subtypes, we observed higher *ECRG4* expression in normal-like cases (*p* = 8.25E-72, one-way ANOVA, **Figure 2**), consistent with a higher expression in NB.

**Table 2:** Correlation of *ECRG4* expression and histoclinical features (n = 1,387).

Characteristics (N)	mean *ECRG4* expression (compared to NB)	*p*-value
Age (years)		6,17E-03
≤50 (380)	−2,6	
>50 (637)	−2,81	
Histological type		7,72E-03
DUC (509)	−2,71	
LOB (32)	−2,28	
MIX (28)	−2,54	
MED (24)	−3,45	
Other (31)	−2,69	
Clinical stage		1,64E-03
I (86)	−2,47	
II-IV (211)	−2,94	
pN		8,69E-03
Negative (460)	−2,6	
Positive (619)	−2,79	
pT		1,30E-03
pT1 (320)	−2,53	
pT2-4 (723)	−2,79	
ER (IHC)		0,47
negative (499)	−2,74	
positive (624)	−2,69	
PR (IHC)		0,11
negative (364)	−2,77	
positive (363)	−2,62	
ERBB2 (IHC)		0,32
negative (261)	−2,75	
positive (100)	−2,88	
SBR grade		1,55E-10
1 (172)	−2,25	
2 (475)	−2,56	
3 (631)	−2,88	
SSP molecular subtype		8,25E-72
Luminal A (419)	−2,62	
Luminal B (188)	−3,33	
Basal (375)	−2,76	
ERBB2 (168)	−3,28	
Normal-like (237)	−1,57	
pCR		0,53
No	−2,6	
Yes	−2,47	

N, number of samples with data available; ILC, invasive lobular carcinoma; MED, medullary carcinoma; IDC, invasive ductal carcinoma; pN, pathological lymph node involvement; pT, pathological tumor size; IHC, immunohistochemistry; ER, estrogen receptor; PR, progesterone receptor; SBR, Scarff, Bloom and Richardson; SSP, single sample predictor [20]; pCR, pathological complete response to primary chemotherapy defined as disappearance of the invasive component of the primary tumor after treatment. HR, hazard ratio; 95CI,95% confidence interval.

**Figure 3:**
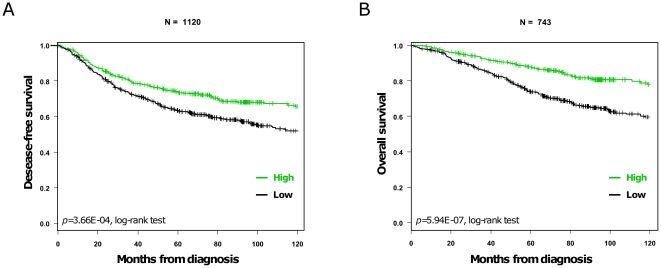
Disease-free and overall survivals according to *ERCG4* mRNA expression. (A) Kaplan-Meier DFS curves in patients with high and low expression (cut-off defined with Cox proportional-hazards regression model built on the IPC data). The respective 5-year DFS are 73 and 63%. (B) Kaplan-Meier OS curves (the legend is similar to A). The respective 5-year DFS are 88 and 74%.

We then examined the prognostic value of *ECRG4* expression in non-stage IV patients. Regarding DFS, the follow-up was available for 1,120 patients (68% 5-year DFS): 365 patients experienced a relapse of their disease after a median time of 24 months from diagnosis, and 755 remained relapse-free with a median follow-up of 70 months. In univariate analysis (**Table 3**), high *ERCG4* expression (HR = 0.84 [0.76-0.92]; *p* = 0.0002), as well as age superior to 50 years, node-negative status, small tumor size (pT1), low grade (SBR 1), positive ER and PR status, and negative ERBB2 status, were associated with a better DFS. **Figure 3A** shows the Kaplan-Meier curves for DFS according to *ECRG4* expression. However, in multivariate analysis, only *ECRG4* expression maintained its prognostic value (*p* = 0.049, **Table 3**). Regarding OS, data were available for 743 patients (80% 5-year OS): 199 of them died of breast cancer after a median time of 46 months from diagnosis, and 544 were alive with a median follow-up of 94 months. In univariate analysis (**Table 4**), high *ECRG4* expression was associated with longer OS (HR = 0.72 [0.63-0.83], *p* = 4.46E-06), as were node-negative status, small tumor size (pT1), low grade (SBR I), positive ER and PR status, and negative ERBB2 status. The OS Kaplan-Meier curves according to *ECRG4* expression are shown in **Figure 3B** **.** Here too in multivariate analysis, *ECRG4* expression was the only significant parameter with an independent prognostic value (*p* = 0.035, **Table 4**), whereas all other classical prognostic factors (age, pathological tumor size, pathological lymph node involvement, pathological grade, ER, PR and ERBB2 expression) lost their prognostic value.

**Table 3:** Disease-free survival (DFS), Cox regression analyses.

		Univariate			Multivariate		
		N	HR [95CI]	*p*-value	N	HR [95CI]	*p*-value
***ECRG4***		1120	0.84 [0.76–0.92]	0.0002	254	0.82 [0.67–0.99]	0.049
**Age**	>50 vs ≤50 years	958	0.78 [0.63–0.98]	0.03			
**Histology**		472		0.151			
	ILC vs IDC		1.22 [0.69–2.15]				
	MED vs IDC		0.50 [0.20–1.22]				
	Mixed vs IDC		0.46 [0.22–1]				
	Other vs IDC		0.94 [0.48–1.85]				
**pN**	positive vs negative	930	2.21 [1.74–2.80]	5.27 E-11	254	1.22 [0.79–1.89]	0.37
**pT**	pT2-3 vs pT1	879	2.56 [1.93–3.40]	6.79 E-11	254	1.28 [0.79–2.05]	0.31
**SBR grade**	2-3 vs 1	1075	2.80 [1.92–4.07]	8.29 E-08	254	1.37 [0.71–2.67]	0.35
**ER**	positive vs negative	943	0.64 [0.51–0.80]	9.42 E-05	254	0.86 [0.40–1.86]	0.71
**PR**	positive vs negative	572	0.67 [0.50–0.88]	0.004	254	1.02 [0.49–2.12]	0.97
**ERBB2**	positive vs negative	310	2.32 [1.58–3.39]	1.64 E-05	254	1.02 [0.56–1.86]	0.94

N, number of samples with data available; ILC, invasive lobular carcinoma; MED, medullary carcinoma; IDC, invasive ductal carcinoma; pT, pathological tumor size; pN, pathological lymph node involvement; ER, estrogen receptor; PR, progesterone receptor; SBR, Scarff, Bloom and Richardson; HR, hazard ratio;95CI,95% confidence interval.

**Table 4:** Overall survival (OS), Cox regression analyses.

		Univariate			Multivariate		
		N	HR [95CI]	*p*-value	N	HR [95CI]	*p*-value
***ECRG4***		743	0.72 [0.63-0.83]	4.476 E-06	254	0.77 [0.60-0.98]	0.036
**Age**	>50 vs ≤50 years	584	0.86 [0.62-1.17]	0.33			
**Histology**		345		0.12			
	ILC vs IDC		0.93 [0.45-1.93]				
	MED vs IDC		0.46 [0.14-1.44]				
	Mixed vs IDC		0.24 [0.06-0.97]				
	Other vs IDC		0.43 [0.14-1.37]				
**pN**	positive vs negative	563	2.76 [2.00-3.80]	6.90 E-10	254	1.36 [0.82-2.25]	0.24
**pT**	pT2-3 vs pT1	518	3.27 [2.17-4.91]	1.22 E-08	254	1.57 [0.88-2.83]	0.13
**ER**	positive vs negative	578	0.57 [0.42-0.78]	4.84 E-04	254	0.91 [0.35-2.36]	0.85
**PR**	positive vs negative	582	0.57 [0.42-0.78]	1.50 E-04	254	0.78 [0.31-1.95]	0.60
**ERBB2**	positive vs negative	323	2.01 [1.33-3.04]	9.70 E-04	254	0.72 [0.34-1.52]	0.38
**SBR grade**	2-3 vs 1	717	3.82 [2.25-6.49]	6.61 E-07	254	1.97 [0.80-4.85]	0.14

N, number of samples with data available; ILC, invasive lobular carcinoma; MED, medullary carcinoma; IDC, invasive ductal carcinoma; pT, pathological tumor size; pN, pathological lymph node involvement; ER, estrogen receptor; PR, progesterone receptor; SBR, Scarff, Bloom and Richardson; HR, hazard ratio;95CI,95% confidence interval.

Finally, we assessed the correlation between *ECRG4* expression and the response to neo-adjuvant chemotherapy in early breast cancer. We analyzed expression data from 166 cases (41 from our own series and 125 from [19]) pre-operatively treated with an anthracycline or an anthracycline/taxane-based regimen. Out of them, 70 displayed pCR after chemotherapy, and 96 did not. *ECRG4* expression was not correlated with pCR (*p* = 0.36, t-test; **Table 2**).

### Biological pathways associated with *ECRG4* expression

Using Significance Analysis of Microarrays, we identified 891 genes differentially expressed between the 50 tumors with the lowest *ECRG4* expression and the 50 ones with highest expression. Most of these genes (n = 800) were overexpressed in the tumors of the last group. Ontology analysis of these 891 genes revealed that *ECRG4* overexpression was correlated with expression of genes associated with axon guidance, protein kinase A signaling, integrin signaling, endocytosis, ephrin signaling, CXCR4 signaling, and the Wnt/β-catenin pathway (**Table S2**).

## Discussion

The *ECRG4* gene, officially named *C2ORF40*, is highly conserved in vertebrates, not in other eukaryotic species, suggesting an important role in vertebrate organisms. Although identified many years ago, the function of the protein encoded by this gene remains unclear, but recent data revealed a potential TSG role in different cancers. To our knowledge, our study is the first one analysing *ECRG4* in normal and cancer mammary tissues.

Through the analysis of more than 350 breast cancers, we show that *ECRG4* is underexpressed in 94% of tumors. Frequent down-regulation has also been reported in cell lines and clinical tissue samples of esophagal, colo-rectal, and prostate carcinomas, and gliomas. Of note, all breast cancer cell lines profiled in our laboratory also showed very low expression of *ECRG4* when compared to HME1, a non-tumorigenic mammary cell line derived from mammoplasty (data not shown). This underexpression of mRNA may be due to genetic or epigenetic mechanisms, as well as decreased mRNA stability. Here, we show that DNA loss, although relatively frequent (18%), cannot explain the high frequency of downregulation. We did not analyze mutations and DNA methylation. No *ECRG4* mutation has been reported in cancers [7], [24]. Epigenetic alterations of the genome such as DNA promoter methylation play an important role in tumorigenesis of various human cancers by silencing TSG [25]. In breast cancer, multiple TSG are hypermethylated and downregulated, including for examples *BRCA1*, *RASSF1A*, *p16*, *FHIT*, and *CDH1* [26]. Promoter methylation can also be observed in normal breast tissue adjacent to invasive carcinomas [27]. The *ECRG4* 5-prime UTR contains multiple cis-acting elements and 16 CpG islands. In esophagal, colo-rectal, and prostate carcinomas, and gliomas, promoter methylation is the main mechanism of *ECRG4* silencing, and treatment with demethylating agents restore gene expression [7]. Promoter methylation was recently evidenced in the MCF7 breast cancer cell line [11]. However, this is a single example, which calls for methylation analysis of more cancer cell lines and tissue samples since it is likely that promoter methylation contributes for silencing *ECRG4* in breast cancer.

The tumor suppressor function of ECRG4 [28] and the cellular consequences of its silencing remain to be investigated in breast cancer. In cell lines of esophageal [7]–[9] and colo-rectal cancer [11] and glioma [8], the overexpression of *ECRG4* inhibits cell proliferation by blocking the G1/S transition of cell cycle, through increase of p21 and p53 protein expression. The inhibition of proliferation was confirmed *in vivo* after injection of *ECRG4*-transfected esophageal cancer cell lines into athymic nude mice, which led to slower tumor growth [7]. Another *in vitro* effect of *ECRG4* overexpression is the inhibition of cell migration and invasion in cell lines from esophageal carcinoma and glioma [8].

Meta-analysis of histo-clinical correlations in our series of more than 1.000 cases further reinforced the idea that *ECRG4* is a candidate TSG in breast cancer. Consistent with growth and migration inhibitory effects, we found significant associations between mRNA expression, the progression stage, and the tumor grade, with higher expression in early stage, in small tumors, in node-negative cases, and in low grade tumors. Similar observation was reported in smaller series of esophageal cancer at the mRNA [6] and protein [7] levels. Importantly, *ECRG4* expression was associated with DFS and OS in both uni- and multivariate analyses. The patients whose tumor expressed higher levels of *ECRG4* mRNA survived longer and without relapse than those with lower levels. A similar correlation was reported in small series of esophageal [6]–[7] and prostate carcinomas [10].

In conclusion, we report the first large-scale analysis of *ECRG4* expression in breast cancer. Our results suggest that *ECRG4* is a candidate TSG in breast cancer. Based on our observations and literature data, we speculate that *ECRG4* underexpression confers growth and migration advantages to breast cancers, leading to poor prognosis. Functional analyses are warranted to confirm this TSG role in mammary oncogenesis. Potential clinical applications are therapeutic and prognostic. Whatever the mechanism of silencing, restoring *ECRG4* expression in the tumor, either by epigenetic therapy or application of recombinant protein, may represent a promising novel therapeutic approach in breast cancer. Furthermore, *ECRG4* expression may help improve the prognostication of disease.

## Supporting Information

Figure S1 **mRNA expression of** ***ECRG4*** **in each data set, and in the pooled data set.** Box plots of *ECRG4* expression are shown for each data set. Expression values are NB-centered. The horizontal black line represents the level of expression of *ECRG4* in the NB sample. For each box plot, median and ranges are indicated. IPC, Institut Paoli Calmettes, UNC, University of North Carolina; n, number of samples analyzed. No significant difference was observed between the different distributions (Anova, p>0.05).
(PPT)

Table S1 **Description of the breast cancer data sets**
(XLS)

Table S2 **Canonical pathways associated with** ***ECRG4*** **expression**
(XLS)
