# Supplementary figures and images for: Down-Regulation of ECRG4, a Candidate Tumor Suppressor Gene, in Human Breast Cancer
Source: PLoS One. 2011 Nov 16;6(11):e27656. doi: 10.1371/journal.pone.0027656 (PMC3218004; doi:10.1371/journal.pone.0027656)

## Slide 1
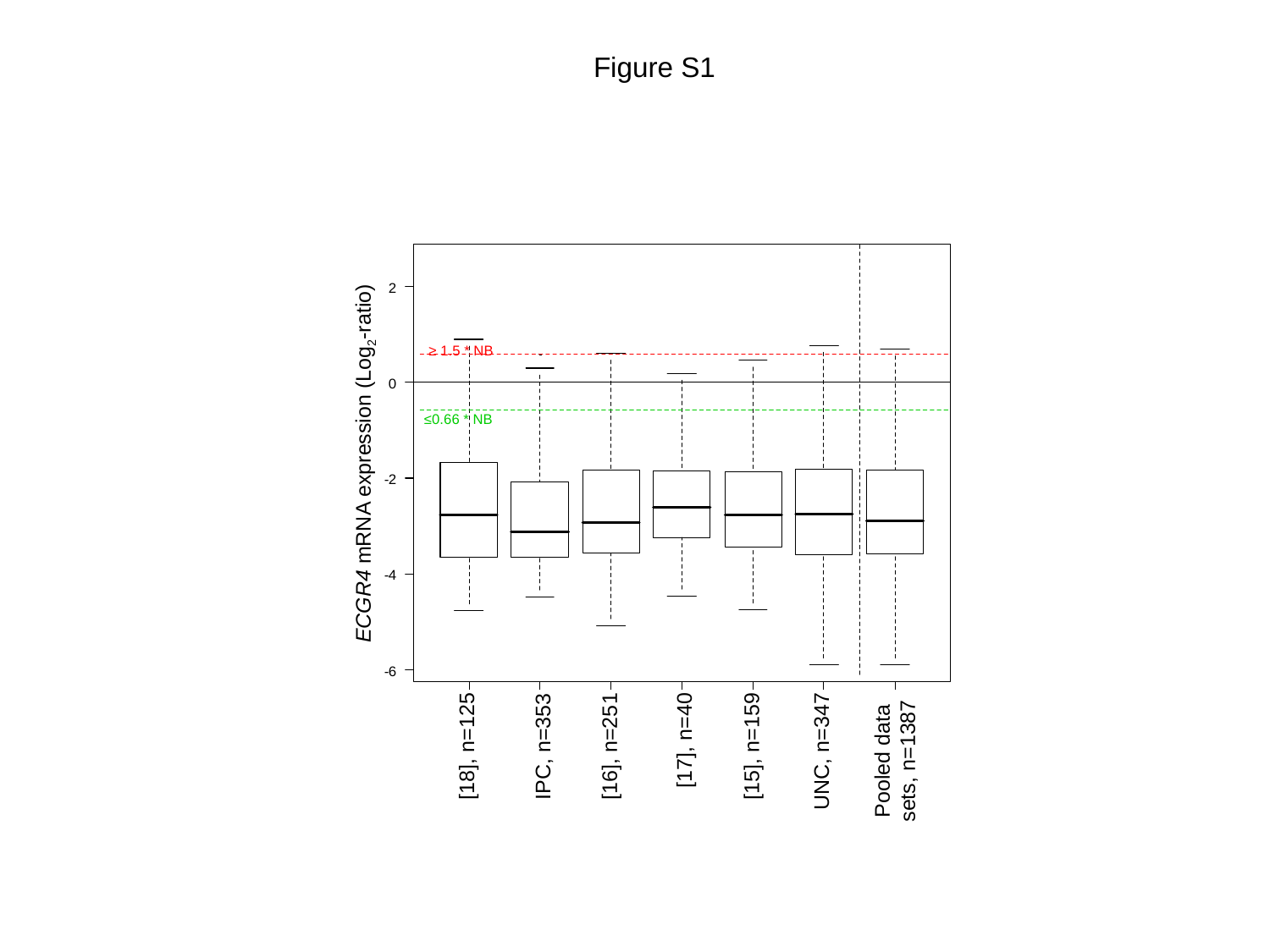

# Figure S1
≥ 1.5 * NB
≤0.66 * NB
ECGR4 mRNA expression (Log2-ratio)
Pooled data sets, n=1387
[18], n=125
IPC, n=353
[16], n=251
[17], n=40
[15], n=159
UNC, n=347

Supplement: Figure S1 — mRNA expression of ECRG4 in each data set, and in the pooled data set. Box plots of ECRG4 expression are shown for each data set. Expression values are NB-centered. The horizontal black line represents the level of expression of ECRG4 in the NB sample. For each box plot, median and ranges are indicated. IPC, Institut Paoli Calmettes, UNC, University of North Carolina; n, number of samples analyzed. No significant difference was observed between the different distributions (Anova, p>0.05). (PPT) [file pone.0027656.s001.ppt]
